# Atomic Structure of Hardening Precipitates in Al–Mg–Si Alloys: Influence of Minor Additions of Cu and Zn

**DOI:** 10.1021/acsnano.3c09129

**Published:** 2023-11-27

**Authors:** Emad H. Bartawi, Calin D. Marioara, Ghada Shaban, Constantinos Hatzoglou, Randi Holmestad, Rajan Ambat

**Affiliations:** ‡ Department of Civil and Mechanical Engineering, Technical University of Denmark, Kgs. Lyngby 2800, Denmark; † Materials and Nanotechnology, SINTEF Industry, Trondheim N-7465, Norway; § Department of Materials Science and Engineering, NTNU, Norwegian University of Science and Technology, Trondheim 7491, Norway; ∥ Department of Physics, NTNU, Norwegian University of Science and Technology, 7491 Trondheim, Norway

**Keywords:** aluminum alloys, recycling, hardening precipitates, crystal structure, HAADF-STEM, APT

## Abstract

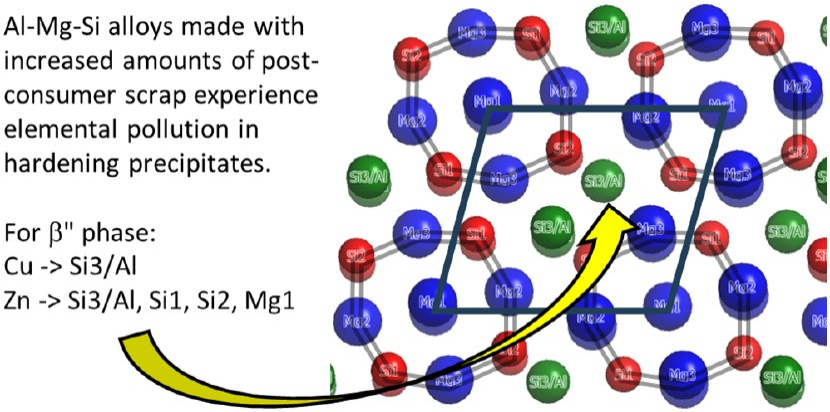

Shifting toward sustainability and low carbon emission necessitates recycling. Aluminum alloys can be recycled from postconsumer scrap with approximately 5% of the energy needed to produce the same amount of primary alloys. However, the presence of certain alloying elements, such as copper and zinc, as impurities in recycled Al–Mg–Si alloys is difficult to avoid. This work has investigated the influence of tiny concentrations of Cu (0.05 wt %) and Zn (0.06 wt %), individually and in combination, on the precipitate crystal structures in Al–Mg–Si alloys in peak aged and overaged conditions. To assess whether such concentrations can affect the hardening precipitate structures, atomic resolution high-angle annular dark-field scanning transmission electron microscopy and atom probe tomography were adopted. The results indicate that low levels of Cu or Zn have a significant influence. Both elements showed a relatively high tendency to incorporate into precipitate structures, where Cu occupies specific atomic sites, creating its own local atomic configurations. However, Zn exhibited distinct behavior through the formation of extended local areas with 2-fold symmetry and mirror planes, not previously observed in precipitates in Al–Mg–Si alloys. Incorporation of Cu and/or Zn will influence the precipitates’ electrochemical potential relative to matrix- and precipitate-free zones and thus the corrosion resistance. Furthermore, the presence of Cu/Zn structures (e.g., β′_Cu_, Q′/C) enhances the thermal stability of these precipitates and, accordingly, the mechanical properties of the material. The results obtained from this work are highly relevant to the topic of recycling of aluminum alloys, where accumulation of certain alloying elements is almost unavoidable; thus, tight compositional control might be critical to avoid quality degradation.

Heat-treatable Al–Mg–Si alloys are intensively used in different applications such as automotive and construction sectors due to their attractive properties, e.g., recyclability, good corrosion resistance, high strength-to-weight ratio, and formability.^1,2^ Using aluminum alloys in automotive applications is an effective strategy for weight reduction without losing performance and thus reducing CO_2_ emissions.^3^ Primary aluminum production requires high energy input due to the electrolysis process. However, recycling can save up to 95% of the energy needed to produce the same amount of primary aluminum, significantly reducing CO_2_ emission.^4,5^ The type and the amount of certain alloying elements that can exist in the final recycled alloys as impurities (∼0.02 at. %) can notably influence their intergranular corrosion (IGC) resistance. Several studies concerning Cu and Zn have reported that Cu (≥0.1 wt %) and Zn (≥0.1 wt %) can significantly influence age hardening response,^6−9^ IGC resistance,^10−12^ and precipitation^7,13−15^ in Al–Mg–Si alloys. However, the interest in investigating the influence of Cu and/or Zn as impurities (∼0.02 at. %) on the IGC resistance of recycled Al–Mg–Si alloys has recently received particular attention^16,17^ due to its importance in recycling. The presence of Cu, which is more noble than Mg and Si, inside the precipitate structure shifts the corrosion potential toward a more positive value, thus influencing the corrosion resistance of the material. However, the presence of Zn, which is less noble than Mg and Si, within the precipitate structure moves the corrosion potential in a negative direction. It is reported that low Cu or Zn contents can influence the corrosion potential of Cu- and Zn-containing particles as well as the corrosion potential differences between the precipitate-free zone, Al matrix, and grain boundaries.^16,18−20^

In the final steps of processing, Al–Mg–Si alloys experience high-temperature exposure during solution heat treatment or during extrusion, followed by rapid cooling and artificial aging. The high temperature (>500 °C) is above the solvus of the system, which after the fast cooling forms a supersaturated solid solution (SSSS). Here the solute elements substitute Al positions on the face centered cubic (FCC) lattice of the Al matrix, together with a high number of quenched-in vacancies. This mix is unstable, and during artificial aging at intermediate temperatures (usually between 150 and 200 °C) the solute atoms will diffuse and cluster into nanosized metastable precipitates, which strengthen the material. These precipitates have one full coherency direction with the Al matrix, along the crystallographic ⟨1 0 0⟩ Al directions, causing them to grow as needles or rods. Along the needle directions the precipitates preserve the structure of FCC Al, meaning that their cross sections consist of two atomic planes separated by *a*/2 = 2.025 Å, each with the periodicity *a* = 4.05 Å of Al. Viewed along the needle direction, the atoms on the two planes are seen as projected atomic columns.^15,21^ Depending on the alloy composition and thermomechanical treatment, the precipitates will have various atomic structures that evolve toward the equilibrium phase of the system. It is commonly accepted that the sequence of precipitation in the Al–Mg–Si system is given as^22,23^
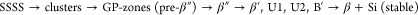
The monoclinic β″ is the phase responsible for the peak hardness, while the hexagonal β′, the trigonal U1, the orthorhombic U2, and the hexagonal B′ form upon overaging. β″ is composed of a structural unit that resembles an “eye” when viewed along the needle direction. This unit can connect in three different ways, forming three different types of β″ phase, labeled as β″ (the most common), β_2_″, and β_3_″.^24^

Several studies have proven that introducing Cu into Al–Mg–Si alloys can significantly enhance their mechanical properties by altering the clustering behavior and improving thermal stability.^9,13,25−31^ For example, Marioara et al.^13^ investigated the influence of Cu addition on the precipitation in Al–Mg–Si alloys aged at 175 °C for different aging times. The results indicated that the alloy with 0.3 wt % Cu exhibited finer microstructure, higher precipitate density, and, thus, higher peak hardness compared to Cu-free alloys. It is also reported that in the peak aged (PA) condition the precipitates comprise only 20–30% of β″ and pre-β′′, while the rest are Guinier–Preston (GP) zones and Q′ precursors, suggesting that β′′ precipitates are not the primary hardening precipitates. Therefore, Cu addition will lead to a more complex precipitation sequence in Al–Mg–Si alloys, which is given as^13,32−34^
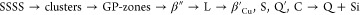
β′_Cu_ has a different atomic structure than the β′ phase in the Cu-free Al–Mg–Si system and is isostructural with the β′_Ag_ phase,^35^ where Cu is occupying the position of Ag.

Recently, investigating the effect of Zn additions on the mechanical properties and precipitation in Al–Mg–Si alloys has become relevant due to the considerable potential of Zn in enhancing age hardening response during artificial aging.^7,29,36−41^ Ding et al.^7^ investigated the impact of Zn additions on the age hardening response in an Al–0.99Mg–0.54Si (wt %) alloy. The results showed that at a relatively high concentration (3 wt %), Zn could enhance the age hardening response by forming GP (II)-zones of η-MgZn_2_ and its precursor. Zhu et al.^29^ reported that introducing Zn into Al–0.9Mg–0.8Si–0.2Cu (wt %) can increase the volume fraction and number density of GP-zones and β″ precipitates, hence enhancing the age hardening response. It was also suggested that Zn additions can increase the partitioning of Mg, Si, and Cu into clusters, enhancing the cluster formation and stimulating the transformation from clusters to precipitates (GP-zones and β″). Xu et al.^38^ reported that a Zn addition up to 2.0 wt % did not change the precipitation sequence in an Al–0.85Mg–1.1Si alloy, while the majority of the intragranular (bulk) precipitates in the alloy containing higher Zn content (4 wt %) consisted of β″ and a small amount of η′ precipitates.

One of the most relevant questions concerning recycling is related to the types and concentrations of scrap-related elements that Al–Mg–Si alloys can tolerate without negatively influencing their IGC resistance and mechanical properties. Also, since such impurities are unavoidable, it is important to know how to avoid the negative influence of the impurities and retain properties similar to primary alloys. In this regard, recent studies on the impact of minor Cu additions (≤0.05 wt %) on IGC resistance as a recycling impurity in 6082–Al–Mg–Si alloy have been reported by Bartawi et al.^16,19^ The results indicated that even such a low Cu addition could cause severe IGC in the PA condition, which is related to the presence of grain boundary (GB) particles and a Cu-rich film along some GBs. Therefore, the current work aims to provide a deep understanding of the influence of 0.05 wt % Cu and 0.06 wt % Zn, individually and in combination, on the precipitate crystal structures. Such information will directly assist in optimizing the mechanical properties through an understanding of the precipitate structures. The IGC resistance can be enhanced by understanding the affinity of Cu and Zn to be incorporated into precipitate structures of Al–Mg–Si alloys and thus moving toward more impurity-tolerant alloys. The affinity and the degree of Cu and Zn incorporation into intragranular and GB particles will inevitably affect the corrosion potential of the Al matrix and the precipitates. Scanning transmission electron microscopy (STEM) and atom probe tomography (APT) were employed in the current work to investigate the precipitate crystal structures and the chemical composition of bulk precipitates on the atomic scale.

## Results

**Figure 1:**
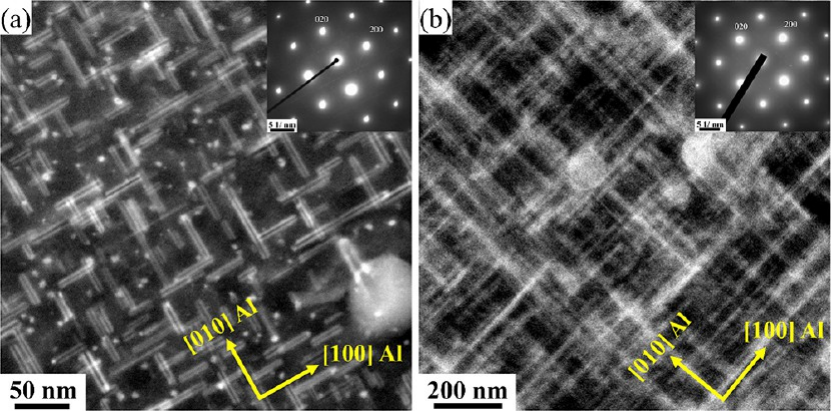
HAADF-STEM images (a, b) of hardening precipitates in alloys O1 and O4, respectively. The corresponding selected area electron diffraction patterns are inserted.

Low-magnification HAADF-STEM images in Figure 1a,b shows the microstructure of alloy O1 with 0.05 wt % Cu in the PA condition and alloy O4 with 0.05 wt % Cu and 0.06 wt % Zn alloy in the overaged (OA) condition, respectively. A fine microstructure with needle-like β″ precipitates in alloy O1 in the PA condition is observed in Figure 1a, while alloy O4 in the OA condition contains a coarser structure with long rod-like precipitates; see Figure 1b. Alloy O2 with 0.06 wt % Zn and alloy O3 with 0.05 wt % Cu and 0.06 wt % Zn in the PA conditions exhibited a similar microstructure to alloy O1; see Figure S1. Based on Figure 1, it is not clear whether Cu or Zn was incorporated into the precipitate structures. Therefore, all alloys were investigated using atomic resolution, atomic number Z-contrast HAADF-STEM imaging to determine the influence of minor additions of Cu (≤0.05 wt %) and Zn (≤0.06 wt %) individually and in combination on the hardening precipitate crystal structures.

Since the precipitates in the Al–Mg–Si system grow along the ⟨0 0 1⟩ Al directions, all the acquired images were taken with the matrix oriented in a ⟨1 0 0⟩ Al zone axis, enabling the visualization of atomic columns in the precipitate cross sections. The acquired images were filtered to reduce scanning noise by applying a fast Fourier transform (FFT) using the band-pass mask (Figure S2), which removed all features that were distanced less than 1.5 Å in real space. To determine the crystal structure of the selected representative precipitates, their projected atomic columns were overlaid with atoms. The overlay was based on Z-contrast of atomic columns, the solved crystal structures of different types of hardening precipitates in the Al–Mg–Si–Cu system,^13,42−45^ and the construction rules of these precipitates.^21,46^ The rules dictate that in the cross-section projection each Al atom in one plane is surrounded by four near neighbor atoms belonging to the other plane, Mg by five, and Si and Cu by three. One consequence of the rules is that the Si atomic columns will have a projected near-hexagonal configuration with an approximately 4 Å separation in the cross-section plane, also called the “Si network”.^13,21,33,44^

Terms such as *pure*, *interface*, *fragmented*, and *mixed* are used in the following to describe the precipitate structures and the influence of minor Cu and Zn additions. *Pure* denotes that the Cu and/or Zn enter the β″ precipitates and partially occupy different Si and/or Al columns without altering their structures. *Interface* refers to the structure in the precipitate at the border between the precipitate and the surrounding Al matrix. *Interfacial* Cu and/or Zn describes a precipitate where, for example, Cu and/or Zn occupy Al matrix columns at the precipitate/matrix interface without entering the precipitate crystal structure. In a *fragmented* precipitate Cu and/or Zn atoms occupy specific local atomic configurations such as in U1, U2, B′, β′_Cu_, and Q′/C phases.^42,47^ Therefore, one such precipitate comprises fragments of different phases in the same needle cross section. *Mixed* precipitates are defined as containing Cu and/or Zn in their bulk (*fragmented*), at the precipitate/matrix interface (*interfacial*), as well as disordered areas that cannot be identified as belonging to known precipitate structures, for example, with 2-fold symmetry and/or mirror symmetries.

### Influence of Cu on the Precipitate Structures in the Peak Aged Condition

**Figure 2:**
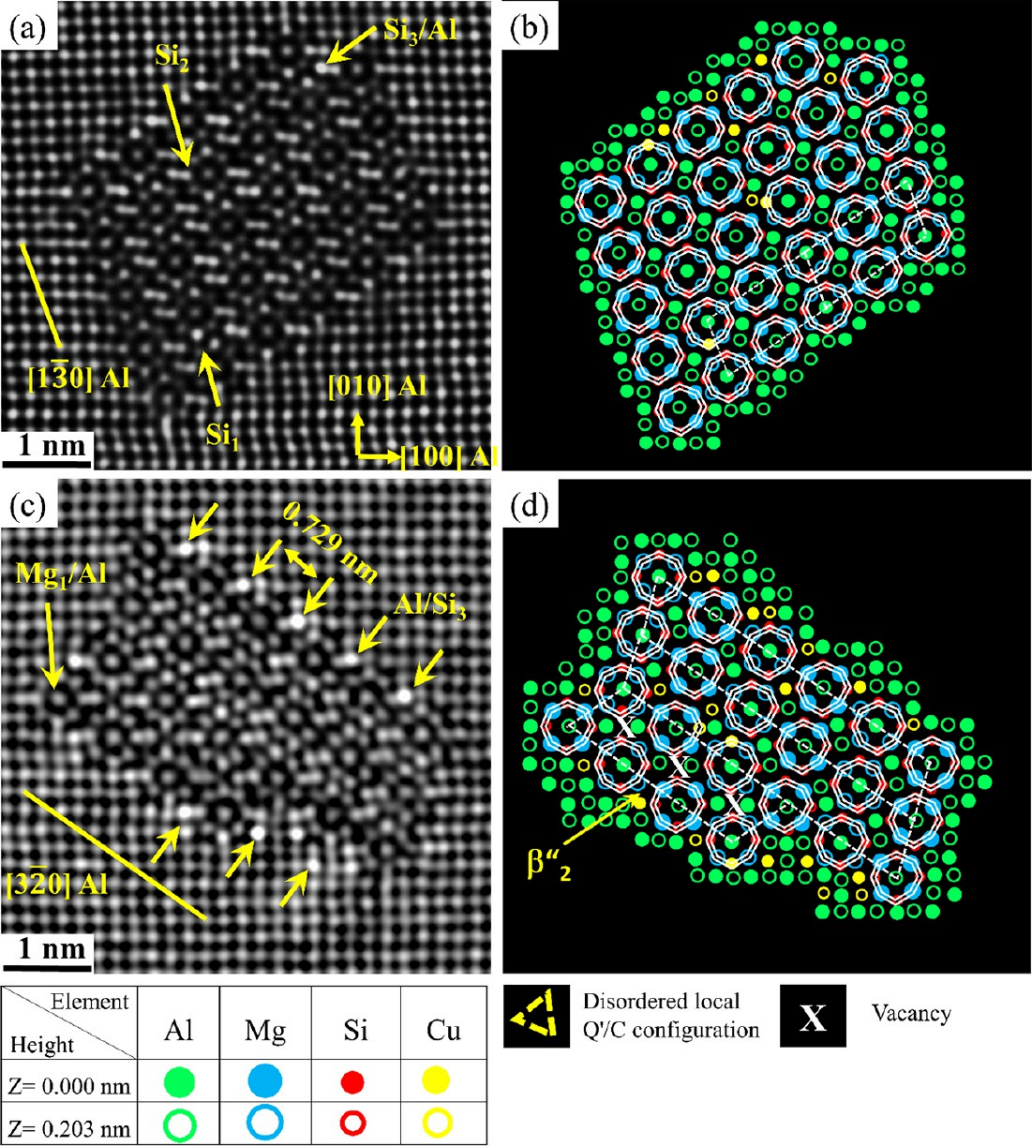
HAADF-STEM lattice images of the cross section of β″ precipitates found in the O1 alloy with 0.05 wt % Cu in the PA condition (a, c) and their suggested atomic overlay (b, d), respectively.

Starting with Figure 2a,c acquired from alloy O1 (0.05 wt % Cu) in the PA condition, β″ precipitates show abnormally brighter columns inside their structures and at the β″/Al interface than neighboring columns. Figure 2 shows brighter Si_1_, Si_2_, and Si_3_/Al columns in a pure β″ structure, indicating that Cu partially occupied these sites.^42,47^ The systematically higher intensity observed at the β″/Al matrix interface corresponds to the Si_3_/Al columns along [3 −2 0] Al; see Figure 2c,d.

**Figure 3:**
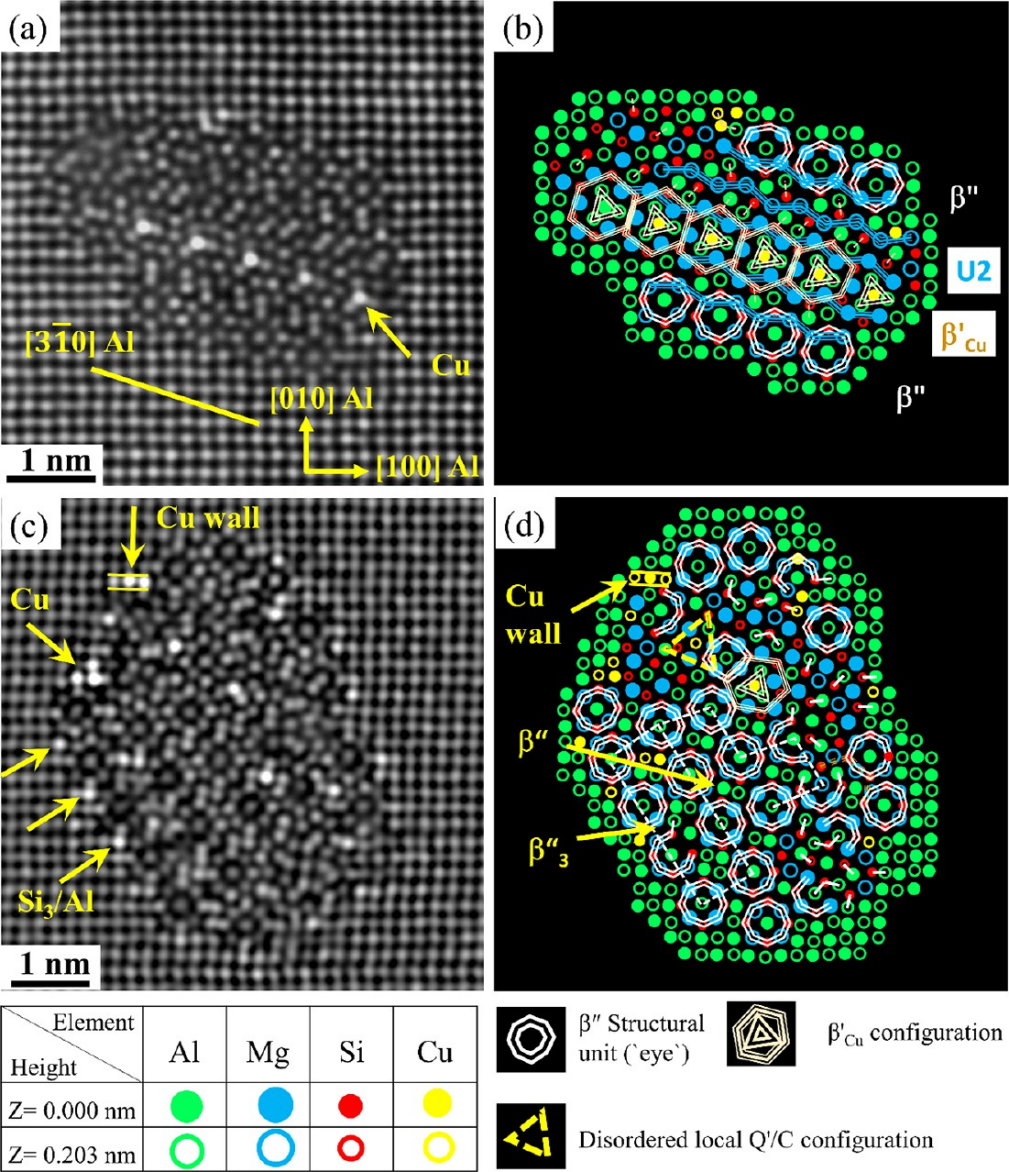
HAADF-STEM lattice images of fragmented precipitate cross sections found in the O1 alloy with 0.05 wt % Cu in the PA conditions (a, c), and (b, d) their suggested atomic overlay, respectively.

Figure 3a,c shows fragmented β″ precipitates that contain several local atomic configurations from known phases (e.g., β′_Cu_, Q′/C, and U2), leading to more complex atomic structures. The β″ and fragmented β″ phases are the primary hardening precipitates observed in alloy O1 in the PA condition. Due to the partial occupancy of Cu in specific columns of the crystal structure of almost all investigated precipitates, those columns exhibit a higher intensity than other equivalent columns. This observation interestingly highlights the significant influence of 0.05 wt % Cu addition on the precipitate structures. Furthermore, it is also noted that no Cu enrichment was observed in the atomic columns of the U2 fragment of the precipitate in Figure 3a,b.

Based on the observation of several precipitates found in the microstructure of alloy O1, the influence of 0.05 wt % Cu on the Al–Mg–Si bulk precipitates can be summarized as follows: ∼22.5% of the acquired precipitates are found to be pure β″ precipitates, ∼23.9% are β″ precipitates with a Cu-enriched interface, and ∼53.5% are fragmented and mixed precipitates; see Table 2. In the fragmented and mixed precipitates, Cu atoms were incorporated into their crystal structure, leading to the emergence of subunits of different phases.

**Table 1:** Chemical Composition (wt %) of 6082–Al–Mg–Si Alloys Studied in the Present Study

alloy no.	Al	Mg	Si	Mn	Fe	Cu	Zn
O1	balance	0.64	0.96	0.54	0.22	0.048	0.003
O2	balance	0.63	0.94	0.56	0.21	0.001	0.059
O3	balance	0.63	0.95	0.58	0.21	0.050	0.059
O4	balance	0.63	0.95	0.58	0.21	0.050	0.059

**Table 2:** Effect of 0.05 wt % Cu and 0.06 wt % Zn, Individually and in Combinations, on the Precipitate Crystal Structures Existing in the Microstructure of the Studied 6082–Al–Mg–Si Alloys in the PA and OA Conditions Using Atomic Resolution HAADF-STEM

Cu/Zn addition	pure β″ (%)	interface (%)	fragmented and mixed (%)	number of investigated cross sections
O1	22.5	23.9	53.5	68
O2	39.8		60.2	74
O3	21.8	3.7	74.5	52
O4			100	33

### Influence of Zn on the Precipitate Structures in the Peak Aged Condition

**Figure 4:**
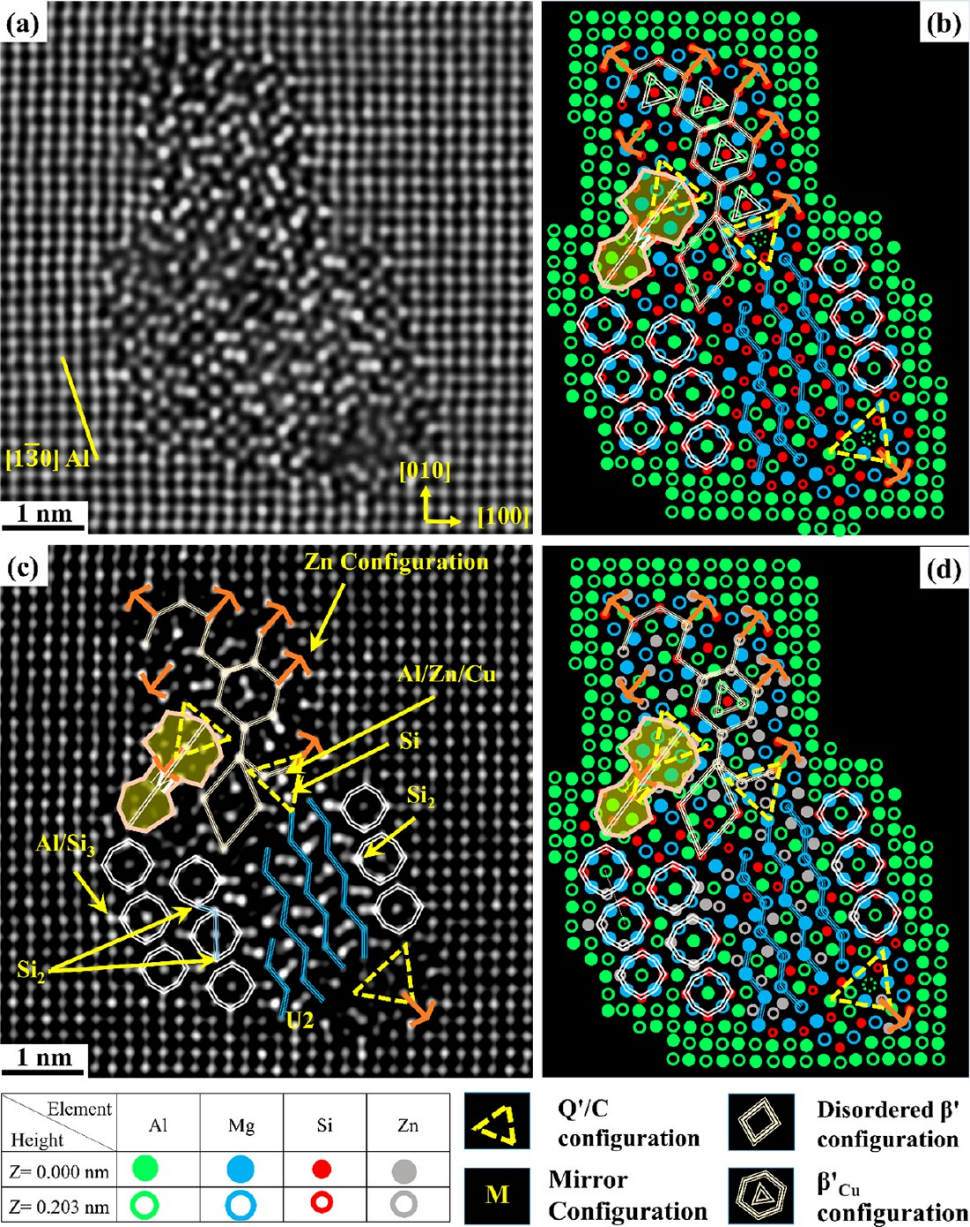
HAADF-STEM images of a Zn-containing β″ mixed precipitate cross section taken from alloy O2. (a) FFT filtered image, (b) suggested atomic overlay based on the construction rules for Al, Mg, and Si, (c) enhanced contrast/brightness image showing different sites and configurations found in this precipitate, and (d) suggested overlay by considering Zn based on Z-contrast.

**Figure 5:**
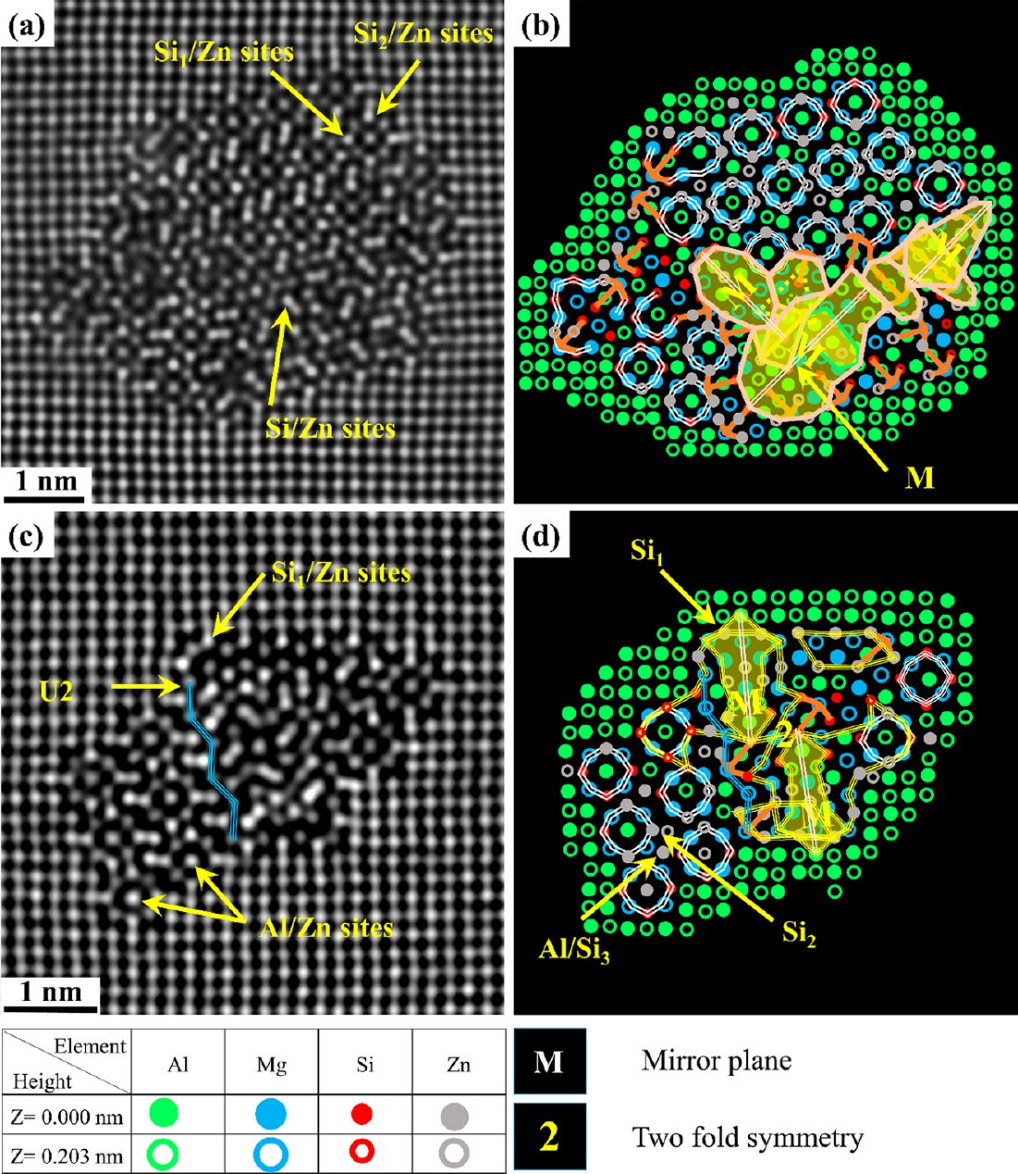
Side-by-side HAADF-STEM images (from O2) of Zn-containing β″ mixed precipitates and their suggested atomic overlay (a–d). Yellow arrows show different atomic sites, e.g., Al, Si_1_, Si_2_, and Si_3_, that are partially occupied by Zn. Two-fold symmetry and mirror plane configurations are shown in (b) and (d).

Figure 4a shows FFT-filtered images of one mixed precipitate from the Zn-containing alloy of O2 in the PA condition. The atomic overlay shows that it consists of a combination of β″, Q′/C, β′_Cu_, and U2 structural configurations. Interestingly, one area containing a mirror plane and many arrow-shaped configurations (drawn in orange) is also observed. Atomic columns with abnormally high Z-contrast can be noticed inside and at the Al matrix/precipitate interface; see Figure 4a,c. Furthermore, extended areas with 2-fold symmetry, mirror plane, and arrow configurations are observed in the disordered regions in-between β″ unit cells in the precipitates shown in Figure 5a–d. It is interesting to note that many of the areas with mirror plane symmetry are related to the arrow configurations, which is believed (based on the current results) to be triggered by the strong Zn tendency to occupy all atomic columns in the β″ structure except for Mg_2_ and Mg_3_ sites. Unlike Cu in the precipitates of the O1 alloy, Zn (O2 alloy) shows a strong tendency to occupy Al and Si columns in the U2 structure; see Figures 3a, 4c, and 5c.

**Figure 6:**
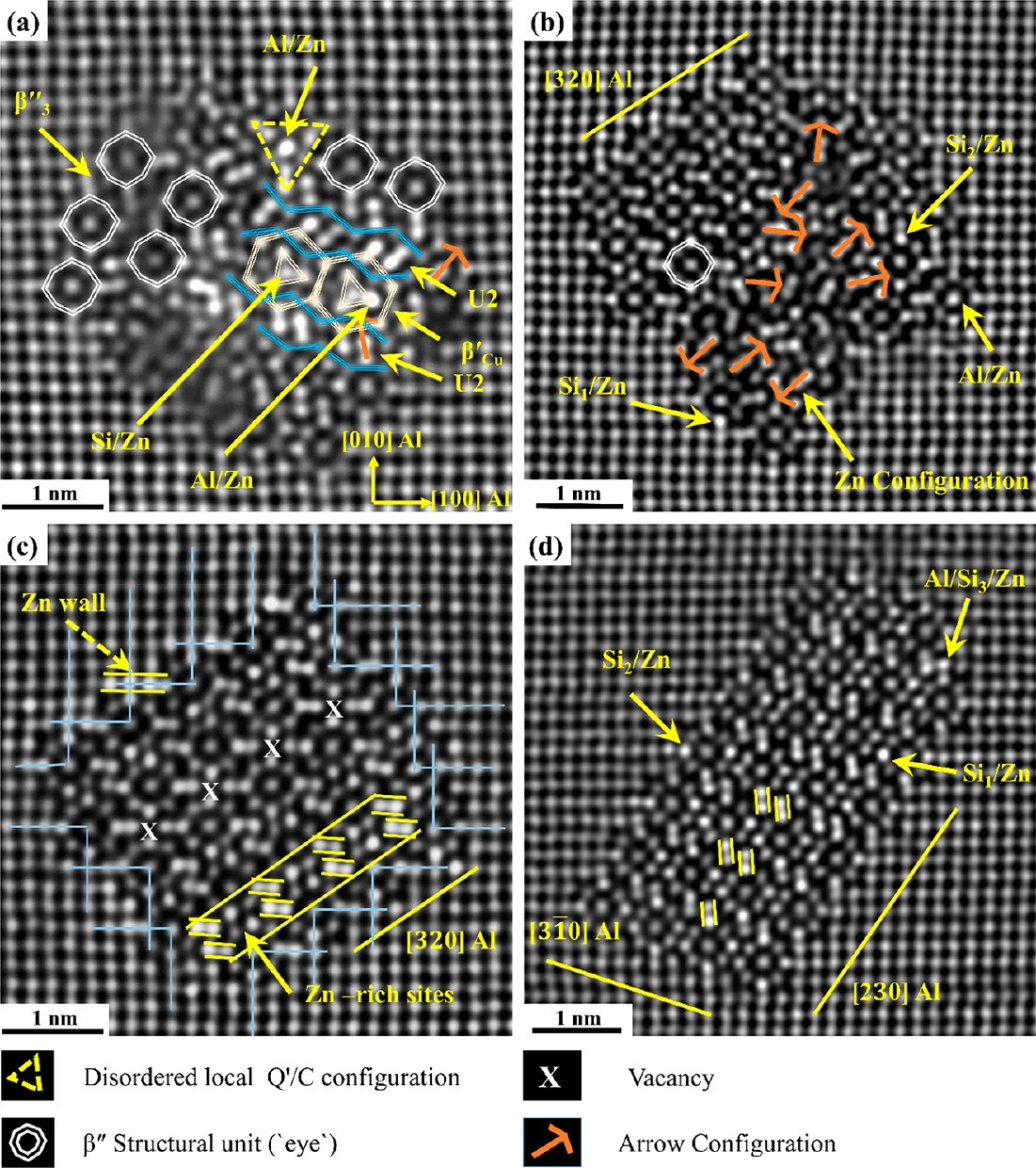
(a, b) HAADF-STEM images from the O2 alloy of Zn-containing β″ fragmented and mixed precipitates, respectively, indicating the local configurations found in their structures, i.e., β″, U2, β′_Cu_, and Q′/C. (c, d) HAADF-STEM images of Zn-containing pure β″ precipitates with yellow arrows showing the atomic sites that Zn has occupied. The dashed yellow arrow shows Zn-occupied Si_2_, Si_3_/Al, and Al sites at the interface, forming a Zn wall usually found in Al–Mg–Cu alloys. Blue solid lines in (c) terminate at Mg_1_/Al sites in β″ precipitates, indicating no interface dislocation (full coherence with Al matrix).

The precipitates in Figure 6 confirm that Zn can enrich Si and Al atomic columns in a multitude of local atomic configurations of β″, U2, Q′/C, and β′_Cu_ phases. In Figure 6a, most of the columns in the layered U2/β′_Cu_/U2 part of the precipitate are Zn-enriched, forming a band with brighter contrast. In addition, Figure 6b, c, and d clearly shows that Zn can be found at the β″/Al matrix interface occupying Si_3_/Al columns and inside the precipitates, occupying different atomic columns in the β″ unit cell. In Cu- and Ag-added Al–Mg–Si alloys, the central Si column in the β′_Cu_ configuration is typically heavily occupied by Cu and Ag atoms. Interestingly, in the O2 alloy, Zn is found to heavily occupy some Al sites more than Si sites; see Figure 6a.

### Influence of Cu and Zn on the Precipitate Structures in the Peak Aged Condition

**Figure 7:**
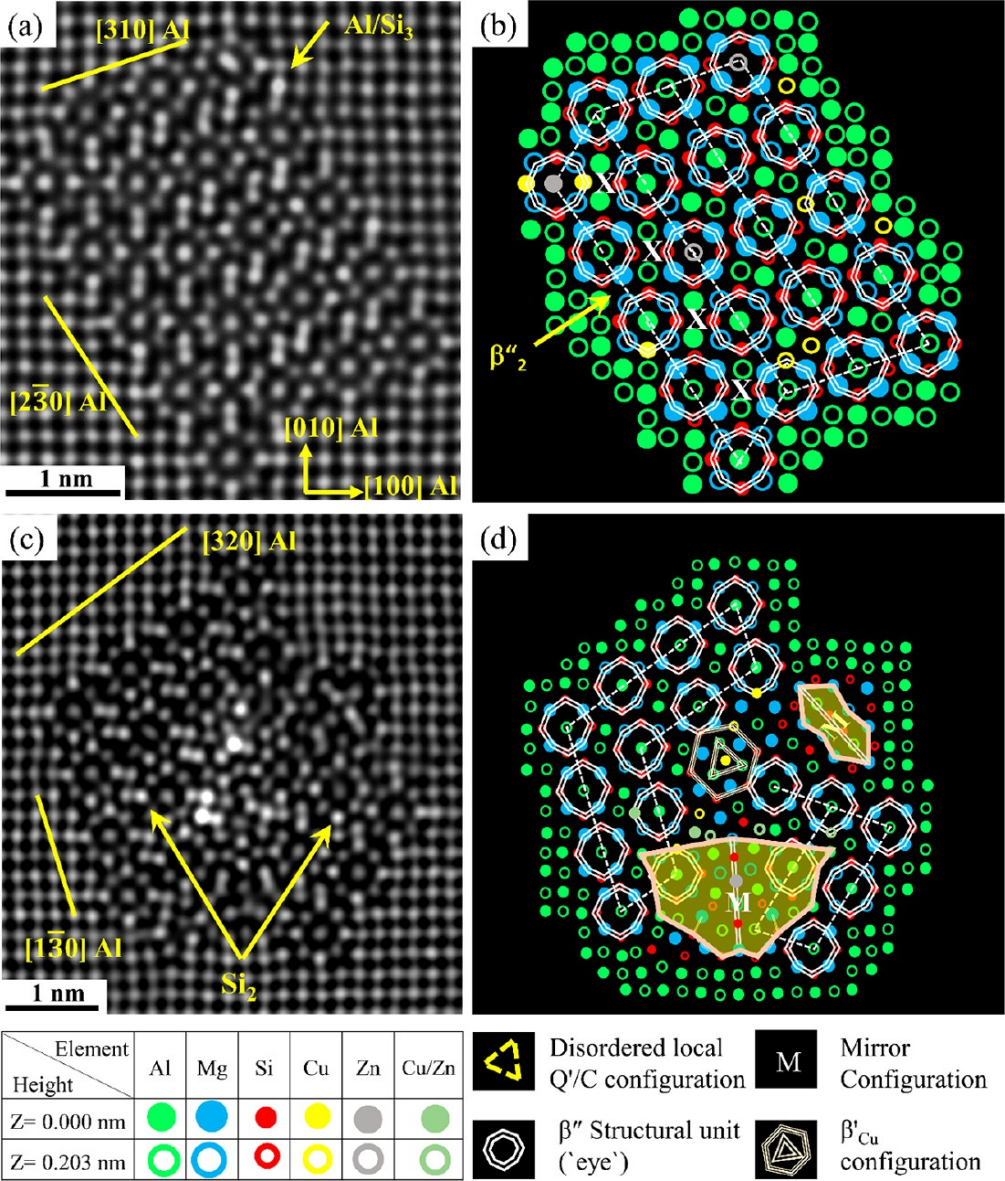
HAADF-STEM lattice images (a, c) of precipitate cross sections from Al–Mg–Si alloy with 0.05 wt % Cu and 0.06 wt % Zn (alloy O3) in the PA condition with the corresponding suggested atomic overlay (b, d), respectively.

**Figure 8:**
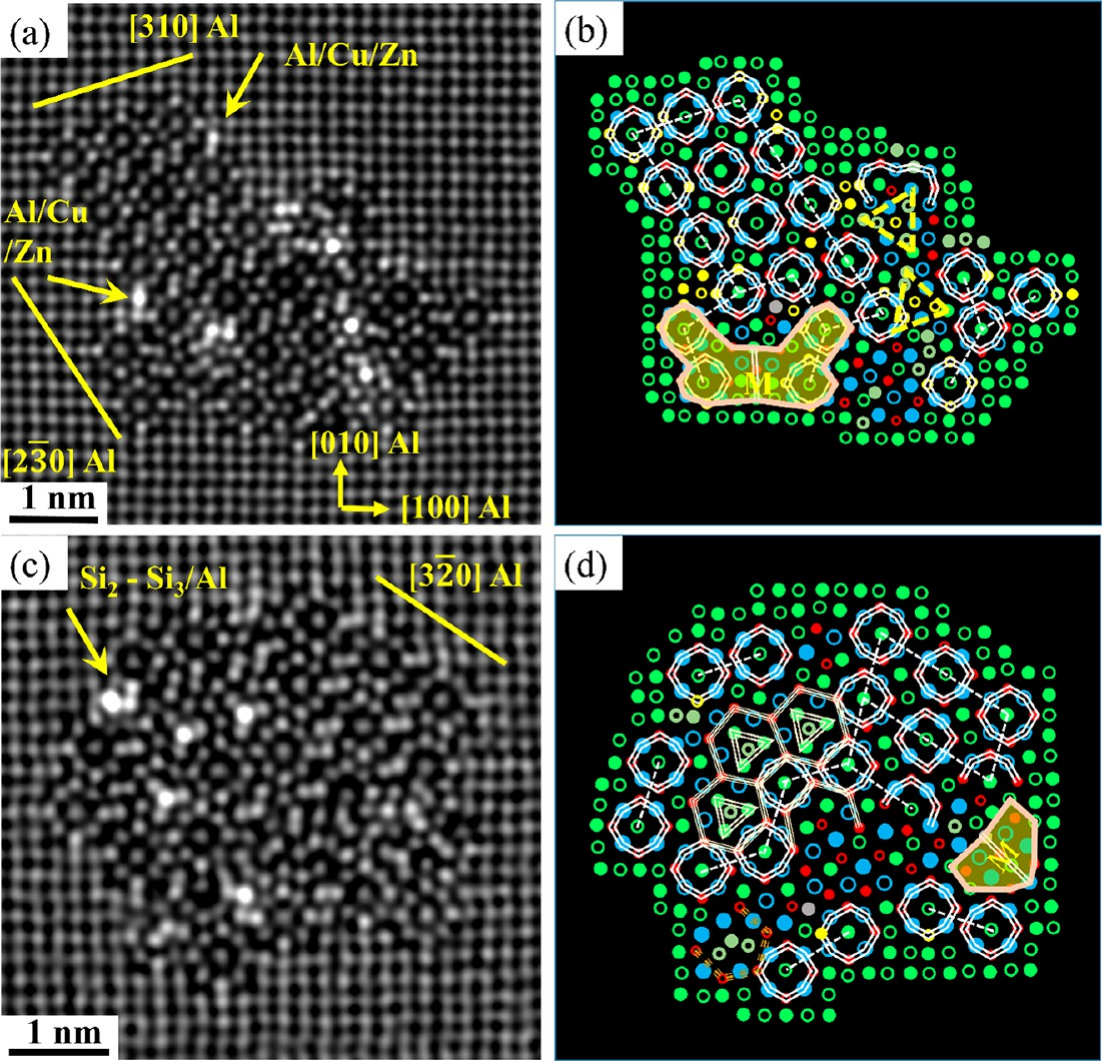
HAADF-STEM lattice images (a, c) of mixed precipitates observed in the microstructure of alloy O3 in the PA condition along with the corresponding suggested atomic overlay (b, d), respectively. See the legend in Figure 7.

Similar to alloys O1 and O2, alloy O3 contains precipitates having a number of atomic columns with higher intensity than other corresponding columns, owing to a partial occupancy of Zn and/or Cu. At the same time, precipitates in alloy O3 reveal evident differences concerning their structures and the fraction of fragmented and mixed precipitates compared to alloys O1 and O2, as shown in Table 2, Figure 7a,c, and Figure 8a,c. Figure 7a,c shows a perfect β″ structure and fragmented β″ precipitates. Atomic columns with abnormally high contrast can be seen inside the precipitate structures, indicating the partial occupancy of Zn/Cu in these columns. It is worth mentioning that the minor Zn and Cu additions lead to the appearance of β′_Cu_ in the fragmented precipitates (Figure 7d) and both β′_Cu_ and Q′/C configurations in the fragmented/mixed β″ crystal structure; see Figure 8b,d. Also, interestingly, mirror planes and arrow configurations are observed in this alloy; see Figure 7d and Figure 8b,d. Since these atomic arrangements have not been observed in alloy O1, it unequivocally demonstrates that the addition of Zn has a distinct impact on the structures of precipitates. The effect of 0.05 wt % Cu and 0.06 wt % Zn additions on the precipitate structures of alloy O3 can be summarized as follows: ∼21.8% of the recorded precipitates are identified as pure β″ precipitates, ∼3.7% are β″ precipitates with Cu/Zn interface enrichment, and ∼74.5% are fragmented and mixed precipitates; see Table 2. Therefore, it can be inferred that significant changes in the fraction of pure and fragmented/mixed precipitates are observed in alloy O3 compared to alloys O1 and O2. Furthermore, the complexity of fragmented/mixed precipitates is observed to be higher in the Zn-added alloys (O2 and O3) compared to only Cu-added alloy (O1); see Figures 3, 4, 5, 6a,b, 7c,d, and 8.

### Influence of Cu and Zn on the Precipitate Structures in the Overaged Condition

**Figure 9:**
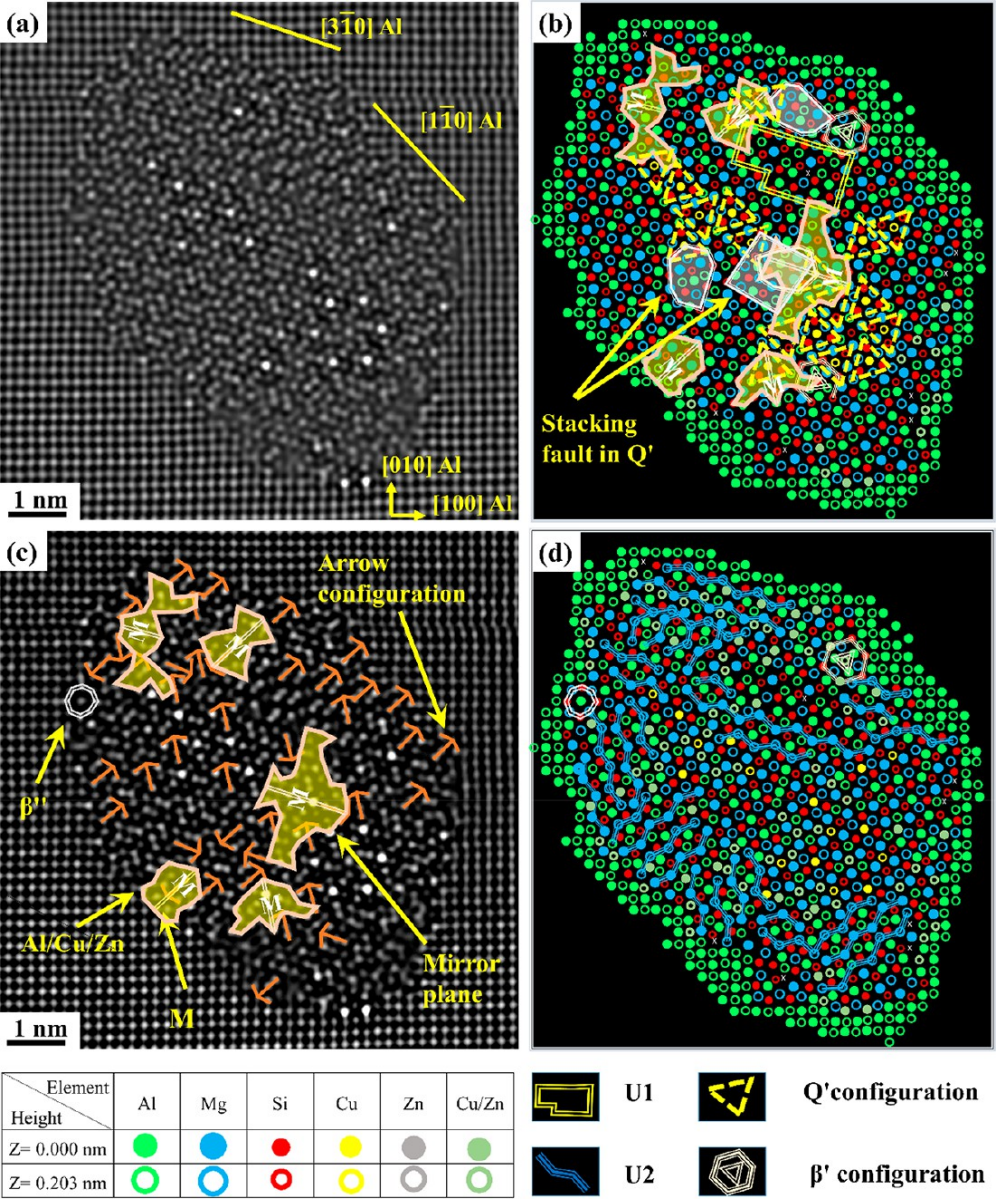
HAADF-STEM images of a mixed precipitate cross section found in alloy O4 in the OA condition. (a) FFT filtered image, (b) suggested overlay based on the construction rules considering Al, Mg, and Si, (c) enhanced contrast/brightness image illustrating different mirror planes and arrow configurations noticed in the precipitate, and (d) suggested overlay by additional consideration of Cu and Zn based on known sites that Cu can occupy and Z-contrast.

**Figure 10:**
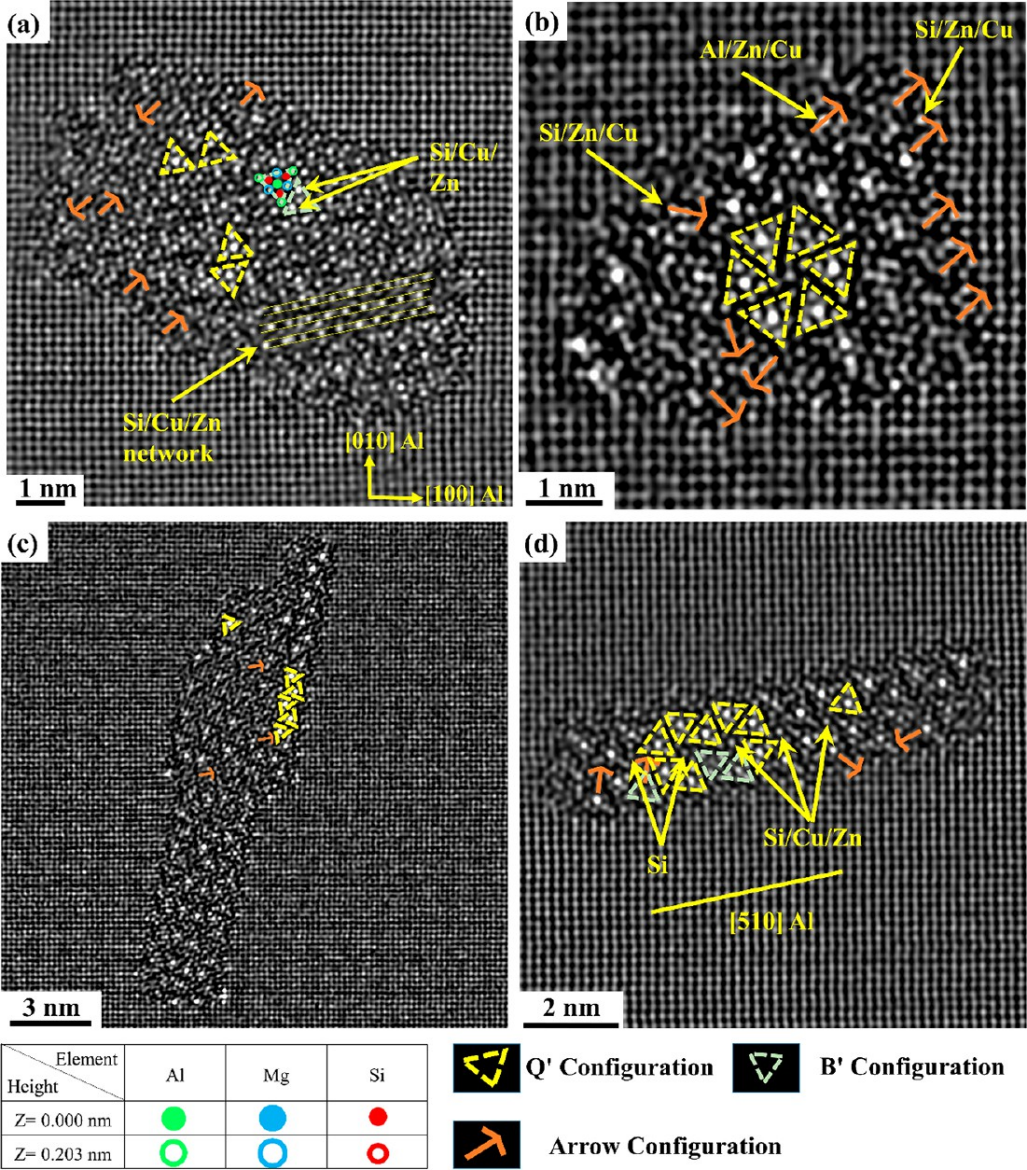
HAADF STEM images of typical precipitate cross sections observed in the microstructure of alloy O4 (a–d), showing the Zn and Cu enrichment of the Q′/B′ phase, Si network, and arrow configurations.

Figure 9a shows the atomic resolution of one of the mixed precipitates observed in the microstructure of alloy O4 in the OA condition along with the suggested atomic overlay considering the construction rules for these precipitates; see Figure 9b. The suggested overlay after also considering the Z-contrast can be seen in Figure 9d. In addition to bright atomic columns, which have been determined to be caused by the presence of Cu, such as in Q′ and β′_Cu_ configurations, bright atomic columns without clear site preferences are also noticed inside the precipitate structures, which pinpoint the effect of Zn. Moreover, mirror planes and several arrow configurations are observed in the precipitates found in this condition; see Figure 9b,c. The amounts of Cu and Zn incorporated into precipitate structures vary. However, all investigated precipitates exhibited a few atomic columns with higher intensity than other corresponding sites; see another example in Figure 10. In general, the precipitates in alloy O4 with 0.05 wt % Cu and 0.06 wt % Zn in the OA condition are of the mixed type and showed the presence of U1, U2, β′_Cu_, Q′/C, β″, and B′ subunit configurations; see Figures 9b and 10.

### Atom Probe Tomography Results

**Figure 11:**
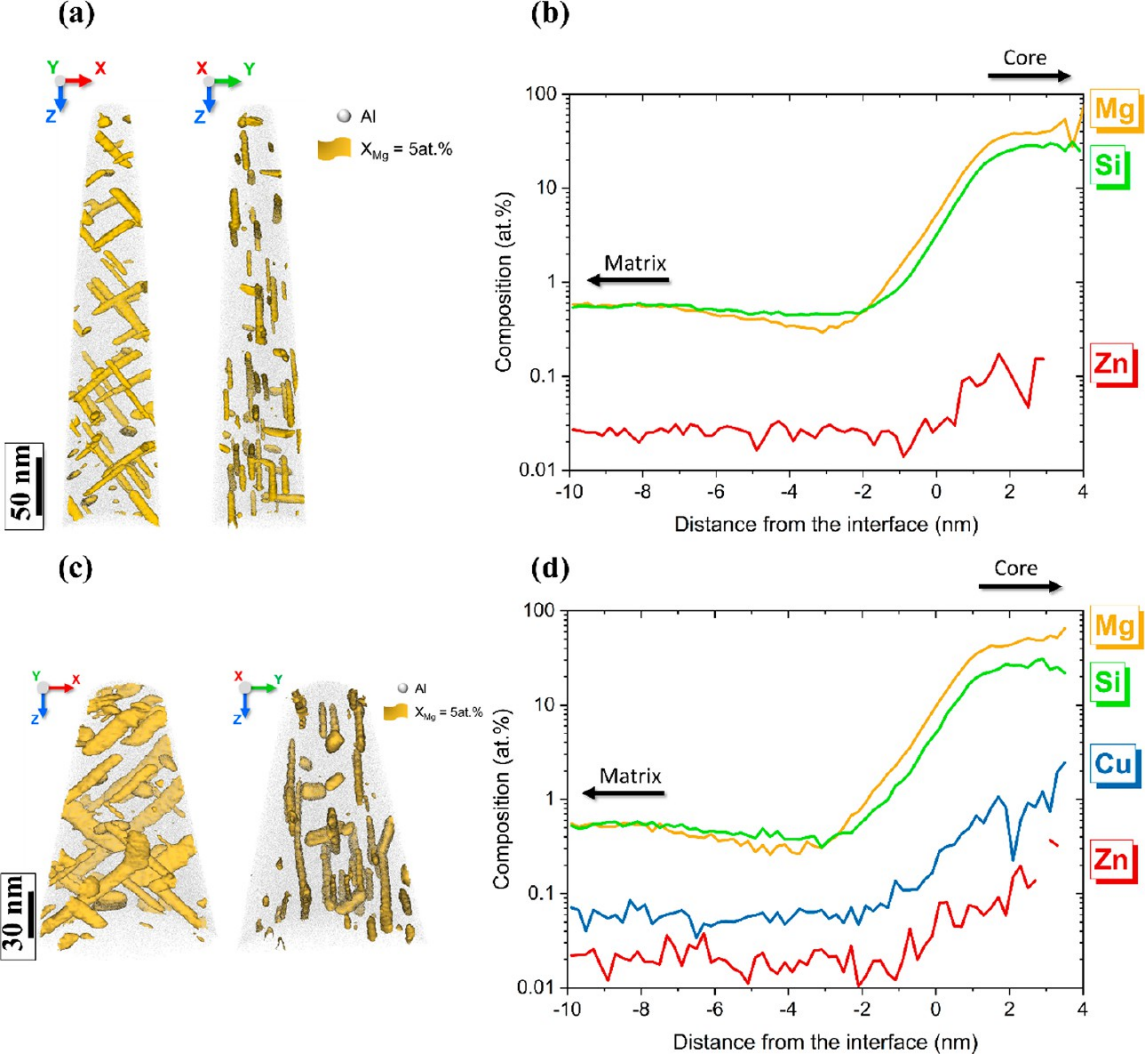
APT analysis of samples from the alloys O2 and O3: (a) Atomic reconstruction of the analyzed volumes displaying the precipitates by Mg iso-concentration surface at 5 at. % from alloy O2. (b) Proximity histogram of all hardening precipitates in the two volumes of the alloy O2. (c) Atomic reconstruction displaying the precipitates by Mg iso-concentration surface at 5 at. % from alloy O3. (d) Proximity histogram of all precipitates found in the two volumes of the alloy O3.

Three samples of alloys O2 and O3 sectioned from the top surface layer of the extruded profiles were investigated by APT, and an example of the volume containing the hardening precipitates is presented in Figure 11. Microstructural features with low volume density such as constituent intermetallic particles, dispersoids, and GB particles are not observed in the evaporated samples from alloy O3. One sample from alloy O2 incorporated a dispersoid (Figure S3). The output from the APT investigations is the distribution of solutes (including Cu and/or Zn) in the microstructure, the average composition of the hardening precipitates (β″ and disordered β″), and the matrix solid solution concentration (between the needles). Concerning the composition of the bulk precipitates observed in alloy O2, the proximity histogram in Figure 11b demonstrates that in addition to Mg and Si they also contain Zn; see Table 3. The latter observation is in agreement with the atomic resolution HAADF-STEM results obtained from alloy O2, as Zn is evidently incorporated into the precipitate crystal structures. The APT results indicate that the bulk precipitates contain ∼0.1 at. % Zn, and their Mg/Si ratio is ∼1.38 ± 0.11. Concerning the composition of the hardening precipitates in alloy O3, the proximity histogram in Figure 11d clearly shows that in addition to Mg and Si they contain Cu and Zn; see also Table 3. The average concentration of Zn in precipitates is ∼0.1 at. %, similar to the alloy O2 case, but the amount of Cu is almost nine times higher, at ∼0.9 at % Cu. Compared to the measured solute concentrations left between needles in the matrix and the nominal alloy composition, these results show that most of the Zn in the alloys stays in solid solution, while the hardening precipitates consume approximately 47% of the Cu added to the alloy O3. The APT results also show that the Mg/Si ratio in the precipitates in the alloy of O3 in the PA condition with 0.05 wt % Cu and 0.06 wt % Zn is 1.77 ± 0.11.

**Table 3:** Nominal, Matrix, and Average Hardening Precipitate Compositions (at. %)^a^

alloy O2 (at. %)				alloy O3 (at. %)			
element	nominal	matrix	precipitate	element	nominal	matrix	precipitate
Mg	0.72	0.29	39.0 ± 1.4	Mg	0.60	0.19	47.3 ± 1.2
Si	0.58	0.31	28.3 ± 1.3	Si	0.44	0.15	26.8 ± 1.0
Zn	0.02	0.02	0.1 ± 0.1	Zn	0.02	0.02	0.1 ± 0.1
Cu	n.s.	n.s.	n.s.	Cu	0.09	0.05	±0.2

a The measurement uncertainties of the nominal and matrix composition are lower than 0.01 at. %. Traces (i*.*e*.*, <0.015 at%) of Ag, Ga, Cr, V, Mn, and Ti are also observed.

## Discussion

The focus in the current work has been to investigate the influence of minor additions of Cu (∼0.05 wt %) and/or Zn (∼0.06 wt %) on the precipitate structures in the PA and OA conditions. Such concentrations can be expected in the final recycled Al–Mg–Si alloys due to the presence of relatively low-quality postconsumer scraps in the recycling process, which drives the need for a better understanding of the impact on the microstructure. Because the HAADF-STEM images display the Z-atomic number, Si atomic columns will have a slightly higher contrast than the Al and Mg columns. It is therefore possible to infer the presence of Cu and Zn by observing which atomic columns have abnormally high contrasts in alloys O1 and O2, respectively. In addition, it is possible to observe the atomic arrangement near these areas to understand if or how Zn and Cu alter the local crystal structure in such concentrations. According to the literature, Cu incorporates into the crystal structure of the hardening precipitates in the Al–Mg–Si–Cu system by occupying certain atomic configurations.^13,31,42^ Consequently, the abnormally brighter columns that do not belong to the solved Cu configurations are presumed to be occupied fully or partially by Zn atoms in the alloys containing both elements (O3 and O4). This assumption is also based on the results obtained from the alloy O2 with only 0.06 wt % Zn addition and on the limited work reported from alloys with a high Zn concentration (1 wt %).^10^ The precipitates presented in the current work are representative of the phases observed in the 6082–Al–Mg–Si alloy in PA and OA conditions with minor addition of Cu and/or Zn, as they are selected from a series of randomly recorded atomically resolved HAADF-STEM images.

### Influence of Cu on the Precipitate Structures in the Peak Aged Condition

Despite the low Cu concentration in alloy O1, Cu incorporated into the structure of β″ and fragmented/mixed precipitates by occupying certain atomic sites, e.g., Si_3_/Al and Si_1_ in β″ precipitates;^47,48^ see Figure 2a,b. The fragmented and mixed precipitates show a complex structure, as at least two of the following subunit configurations are observed in their crystal structures: β″, β′_Cu_, Q′/C, and/or U2; see Figure 3b,d. These outcomes clearly demonstrate the impact of 0.05 wt % Cu on the precipitate structure in the Al–Mg–Si alloy in the PA condition. The influence of Cu on reducing the misfit dislocations at the β″/Al matrix interface has been investigated in an Al–Mg–Si alloy with 0.09 wt % Cu in the PA condition by Saito et al.^49^ It is claimed that the periodic distribution of Cu at the β″/Al matrix interface along ⟨2 3 0⟩ Al can suppress the misfit dislocations observed in Cu-free alloys. Interestingly, a similar effect is noticed in the current work with low Cu content, as the periodic distribution of brighter columns at the β″/Al matrix interface along [3 −2 0] Al suppresses misfit dislocations; see Figure 2c. In a separate work, Sunde et al.^47^ studied the influence of 0.03 and 0.09 wt % Cu addition on the precipitate structures in an Al–0.7Mg–0.9Si (wt %) alloy in different aging conditions. The results showed that most of the precipitates in the PA condition were β″ precipitates. Also, the incorporation of Cu into the β″ structure was higher in the 0.09 wt % Cu added alloy than in the one with 0.03 wt % Cu, leading to the formation of β′_Cu_ subunit configurations in the former. Furthermore, the β′_Cu_ subunit configuration was not observed in the alloy containing 0.03 wt % Cu in the PA condition. Intriguingly, the β′_Cu_, Q′/C, and U2 subunit configurations are observed in this study in the alloy O1, extending the critical Cu concentration that can influence the precipitate structure in the PA condition. Saito et al.^50,51^ reported that Cu was never observed inside the disordered part of a β″ precipitate, as it was only observed within the non-β″ disordered parts or at the β″/matrix interface. In the current work it is evident that Cu partially occupies Si_3_/Al inside the β″ structure found within the mixed/fragmented precipitates and at the β″/Al matrix interface; see Figure 3c,d.

### Influence of Zn on the Precipitate Structures in the Peak Aged Condition

**Figure 12:**
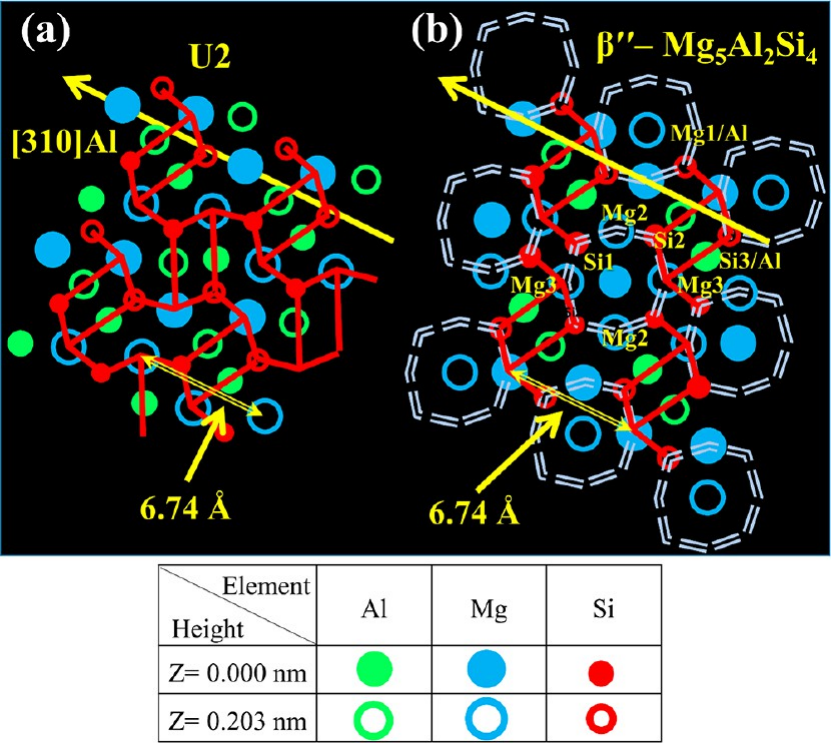
Schematic image showing the arrow configuration as a part of pure β″ and U2 phases.

Saito et al.^10^ investigated the effect of high Zn concentration (1.02 wt %) on the precipitate structure in a leaner Al–0.47Mg–0.39Si (wt %) alloy. The results showed that small amounts of Zn were incorporated into precipitate structures, which led to the formation of disordered precipitates. In the current work, approximately 60.2% of the recorded precipitates in alloy O2 were fragmented and mixed, which comprised β″ subunit structures in combination with subunit structures of other precipitate phases normally found in Al–Mg–Si–Cu alloys, such as U2, β′_Cu_, and Q′/C; see Figures 4b, 5c, and 6a. The remaining 39.8%, were pure β″ precipitates, showing brighter atomic columns in the core part or at the Al matrix/precipitate interface as a result of Zn incorporation; see Figure 6c,d. Z-contrast of atomic columns in such precipitates indicates that the Zn concentration is low. However, Zn atoms show a tendency to randomly and partially occupy Mg_1_/Al, Si_1_, Si_2_, and Si_3_/Al atomic sites; see Figures 4, 5, and 6. Incorporation of Zn into the Si_3_/Al sites is experimentally supported by Saito et al.,^52^ who investigated the influence of high Zn concentration on the precipitate structures in an Al–0.52Mg–0.38Si–0.42Zn (wt %) alloy. The incorporation of Zn into Si_3_/Al, Si_2_, and Si_1_ columns of the β″ unit cell was also supported by density functional theory (DFT) calculations. It has been predicted that Zn atoms are preferentially incorporated in the bulk of the β′′ structure, i.e., not close to the interface. In the same work, Saito et al.^52^ reported that no Zn on the Si_1_ atomic sites was observed experimentally, and Si_2_ had slightly higher intensity attributed to cross-talk artifacts or electron channeling.^52^ The current work evidently shows that Zn can occupy Si_1_ and Si_2_ columns in the bulk of the β″ structure, but no Zn was found in the Mg_2_ and Mg_3_ sites, confirming the DFT calculations. Based on this work, it is believed that the affinity of Zn to occupy Mg_1_/Al, Si_1_, Si_2_, and Si_3_/Al sites in the β″ unit cell caused the formation of an increased number of 2-fold and mirror symmetries in disordered precipitates and the arrow configurations, which are part of the pure (ordered) β′′ and U2 phases; see Figure 12. Interestingly, Zn is also found to occupy the Al and Si sites in the U2 configuration.

The gradual incorporation of Cu into Si_3_/Al and Si_1_ columns of the β″ unit cell has been thought to be the starting point of the formation of Q′/C and β′_Cu_ structural subunits.^47^ The observation of β′_Cu_ and Q′/C substructures without the presence of Cu indicates that Zn atoms can have a similar effect to that of Cu on Al–Mg–Si precipitate crystal structures. Therefore, the observations made in this study suggest that Zn can partially occupy the columns that are normally associated with Cu occupancy in the Q′/C and β′_Cu_ structures. Interestingly, the current results also indicate that Zn can be (partially) occupying Al columns in the β′_Cu_ unit cell, Si columns in Q′/C, and Al and Si columns in the U2 unit cell (found in the fragmented/mixed precipitates); see Figures 4c, 5c, and 6a. The fact that Zn can also occupy the Al sites in the β′_Cu_ makes this phase distinct from β′_Ag_ and β′_Cu_, where the only difference was the identity of the Ag position in the unit cell. For this reason, from here on, in the case of Zn-containing O2 alloy, the β′_Cu_ phase will be referred to as β′_Zn_. All three phases (β′_Ag_, β′_Cu_, and β′_Zn_) are isostructural, with differences in composition and column occupancy. In the case of β′_Ag_ and β′_Cu_, only the center position is occupied by Ag and Cu, respectively, while in the case of β′_Zn_, both the center position and the Al columns can be occupied by Zn. In addition to the above-mentioned, Zn can partially occupy various atomic sites in substructures of the Q′/C, β′_Zn_, and U2 phases and in β″ precipitates. Zn is also observed to have a more distinct effect on precipitate crystal structures that, to the best of the authors’ knowledge, have not been described previously; see Figures 4c, 5b,d, and 9a,b. It promotes the formation of defects with extended 2-fold symmetry and mirror plane configurations, which is not observed in precipitates nucleated in pure Al–Mg–Si alloys or with Ag, Li, and Cu additions.^10,42,57−59,44,49,50,52−56^ Moreover, Zn shows less tendency than Cu to occupy the β″/Al matrix interface periodically, but Zn incorporates preferentially on the Si_2_, Si_3_/Al sites of the β″ structures, which also suppresses misfit dislocations; see Figure 6c,d. It is interesting to note that Zn suppressed misfit dislocations even in the precipitates comprised of β″ and β′′_2_ structures; see Figure 6a. Furthermore, based on the suggested atomic overlay and unlike Cu, once Zn enters the precipitate structure, it can occupy all atomic columns except for Mg_2_ and Mg_3_ sites, which is believed to disturb the Si network and lead to more complex structures; see Figures 4, 5, and 6.

### Influence of Cu and Zn on the Precipitate Structures in the Peak Aged and Overaged Conditions

By studying 0.1 wt % Cu and ∼1 wt % Zn additions in an Al–Mg–Si alloy separately, Saito et al.^53^ showed that the majority of the hardening precipitates lacked an overall periodicity. Moreover, the Zn and Cu columns formed certain local symmetries connected to the Si network. It is also reported that Zn and Cu atoms can occupy Al sites at the precipitate/matrix interface. The presence of Zn in the precipitate structures in alloy O3 cannot be separated using Z-contrast alone, but brighter atomic columns were observed without any clear site preference; see Figures 7 and 8. This is most likely related to the partial incorporation of Zn, as Cu in Zn-free alloys always has a strong site preference associated with β″ Si_3_/Al, Q′/C, and β′_Cu_ configurations.^47,49,50,53,60^ Despite the very low Zn concentrations in the investigated alloys, the current work provides a deeper understanding of the influence of Zn on the precipitate structures, as brighter atomic columns, which do not belong to known unit configurations, are observed in the core part of many precipitates; see Figures 7c and 8c. Furthermore, a noticeably high number of brighter Si_2_ atomic columns (in the β″ structural unit) are observed after Zn additions, indicating that Zn partially occupies such columns; see Figure 7c. Therefore, this study shows that Cu and Zn atoms in combination can have a considerable influence on precipitation in Al–Mg–Si alloys. Interestingly, the quantitative APT measurements of the chemistry of the bulk precipitates in alloy O3 indicate that the affinity of Cu to enter the crystal structure of the hardening precipitates is higher than that for the Zn. Therefore, the amount of Cu in the hardening precipitates is found to be notably higher than that for Zn, even though Zn has a slightly higher concentration in the alloy; see Figure 11d. The Z-contrast of the atomic resolution HAADF-STEM images from alloys O1 and O2 shows a high tendency for Cu and Zn to incorporate into a precipitate structure. Based on these observations, it can be concluded that the occupancy level (fraction of Cu and Zn atoms in the columns) for Cu is higher than that for Zn.

The transformation of the precipitate structures after 5 h of aging at 240 °C (OA condition) in alloy O4 is demonstrated in Figures 9 and 10. The high Z-contrast inside the precipitate structures indicates Cu and/or Zn incorporation; see Figures 9c and 10. The suggested atomic overlay in Figure 9b,d shows a more complicated structure compared to precipitate structures found in alloy O3 (the PA condition) as several mirror-plane and arrow configurations, U1, Q′/C, β′_Cu_, U2, and interestingly β″ structures, are observed. In addition, it is noticed that the Si network in disordered parts of the precipitates in this condition is notably disordered. The presence of the mirror plane configurations in disordered areas is believed to be due to the presence of Zn in this alloy as, to the best author’s knowledge, such configurations are not observed previously in overaged alloys with Cu additions.^60,61^

## Conclusions

The influence of 0.05 wt % Cu and 0.06 wt % Zn additions, individually and in combinations, on the precipitate crystal structures in a 6082–Al–Mg–Si-type alloy was investigated using aberration-corrected HAADF-STEM and APT. The main findings of the current work are summarized as follows:

- Minor additions of Cu and/or Zn have a significant influence on the precipitate structures in Al–Mg–Si alloys in the peak aged and overaged conditions. This indicates that both elements have a high tendency to enter precipitate structures, affecting the electrochemical potential and the thermal stability of the hardening precipitates during heat treatment.
- Cu is observed to occupy Si_3_/Al sites inside β″ structures found within the mixed precipitates as well as periodically occupying the precipitate/Al matrix interface, indicating that even low Cu levels, an impurity commonly observed in recycling, can influence precipitate structures.
- Zn noticeably disturbs the Si network locally within the fragmented/mixed precipitates containing mirror plane symmetries. This effect is not observed when only Cu is added, indicating that the low Zn content in recycled alloys can noticeably alter the precipitate structures, introducing different phases not typically seen in Zn-free alloys. These structures may enhance the thermal stability of recycled alloys.
- Zn shows a high tendency to incorporate into precipitate structures, leading to formation of different resolved subconfigurations found in the Al–Mg–Si–Cu system. Also, it is evident that Zn has a strong tendency to occupy all atomic sites inside the β″ structure except for Mg_2_ and Mg_3_ sites, which is believed to be the reason behind the formation of extended areas with 2-fold symmetry and mirror plane configurations in the mixed precipitates.
- The suggested atomic overlays indicate that Zn does not show a periodic distribution inside the fragmented/mixed precipitate crystal structures, as once entering the structure it can partially occupy all atomic columns (Al, Si, Cu) except for the Mg sites.
- Cu and Zn additions have a significant impact on the precipitate structures in the overaged condition, as U1, U2, Q′/C, β′_Cu_, β′_Zn_, and β″ configurations are observed, in addition to the tendency of the two elements to partially occupy atomic columns of the Si network.
- APT results indicate that Zn has less tendency to incorporate into precipitate structures than Cu, with most of the Zn still found in the solid solution.

## Experimental Methods

### Material Used

The chemical compositions of the Al–Mg–Si alloys used in the current research are displayed in Table 1. The homogenization process of the ingots was conducted at 575 °C for 135 min. A ram speed of 5.6 mm/s was adopted during the hot extrusion process. The average billet temperature during the extrusion was approximately 530 °C, and the final extruded flat profiles had a thickness of 4 mm. Thereafter, the material was water-quenched, stretched to 0.5%, and finally exposed to a two-step artificial aging process to reach the PA condition. Hydro Aluminum refers to this condition as the T6 condition, which is used in this paper as the designation for “as received material”. To investigate alloy O3 in the overaged condition, the extruded alloy was solution heat treated for 30 min at 530 °C, water-quenched, and then artificially aged for 5 h at 240 °C. The OA alloy was denoted as alloy O4; see Table 1.

### Microstructure Characterization

For atomic resolution, high-angle annular dark-field scanning transmission electron microscopy (HAADF-STEM) specimens from each alloy were cut to ∼400 μm thickness, mechanically polished to ∼100 μm, and eventually punched into 3 mm discs. The thin foils were prepared using the TenuPol-5 twin-jet polishing system in a solution containing 900 mL of ethanol and 100 mL of 65% perchloric acid at ∼−20 °C. A spherical aberration (Cs) double-corrected JEOL ARM200F microscope operated at 200 kV was used to acquire the atomic resolution HAADF-STEM images of the bulk precipitates. The HAADF detector had a collection angle range of 51 to 203 mrad, and the probe size was 0.10 nm.

Three specimen tips from each of the O2 and O3 alloys were prepared for APT by electropolishing according to the methods and using solutions described in refs (62) and (63). The APT analysis was done with a CAMECA LEAP 5000XS, operated in UV laser pulsing mode with a frequency of 200 kHz and a detection rate of 2%. The base temperature was set at 40 K, and the laser energy was adjusted to obtain an equivalent pulse fraction of 25% of the DC voltage to avoid preferential evaporation^64^ (i*.*e., around 100 pJ). Data reconstruction and processing were performed using the AP Suite 6.1 software tool and the Norwegian Atom Probe App (NAPA) software, developed in MATLAB.^65^ The APT results for each alloy are a synthesis of three separate evaporated volumes.

## Data Availability

The raw/processed data required to reproduce these findings cannot be shared at this time as the data also form part of an ongoing study.

## Supporting Information Available

Microstructure of O2 and O3 alloys with different Zn content; example showing raw HRTEM image along with the filtering process adopted in this study to reduce the scanning noise; APT analysis showing a dispersoid that can be found in Al–Mg–Si alloys (PDF)
