# Supplementary material for: Atomic Structure of Hardening Precipitates in Al–Mg–Si Alloys: Influence of Minor Additions of Cu and Zn
Source: ACS Nano. 2023 Nov 27;17(23):24115–29. doi: 10.1021/acsnano.3c09129 (PMC10722592; doi:10.1021/acsnano.3c09129)
Supplement: Supplementary file 1 — nn3c09129_si_001.pdf [file nn3c09129_si_001.pdf]

Supporting Information- Atomic structure of hardening precipitates in Al-Mg-Si alloys: Influence of minor additions of Cu and Zn

Emad H. Bartawi[1],*, Calin D. Marioara[2], Ghada Shaban[1], Constantinos Hatzoglou[3], Randi Holmestad[4], Rajan Ambat[1]

[1] *Department of Civil and Mechanical Engineering, Technical University of Denmark, Kgs. Lyngby 2800, Denmark*

[2] *Materials and Nanotechnology, SINTEF Industry, Trondheim N-7465, Norway*

[3]*Department of Materials Science and Engineering, NTNU, Norwegian University of Science and Technology, Trondheim 7491, Norway*

[4]*Department of Physics, NTNU, Norwegian University of Science and Technology, 7491 Trondheim, Norway*

*Corresponding author: ehaba@dtu.dk

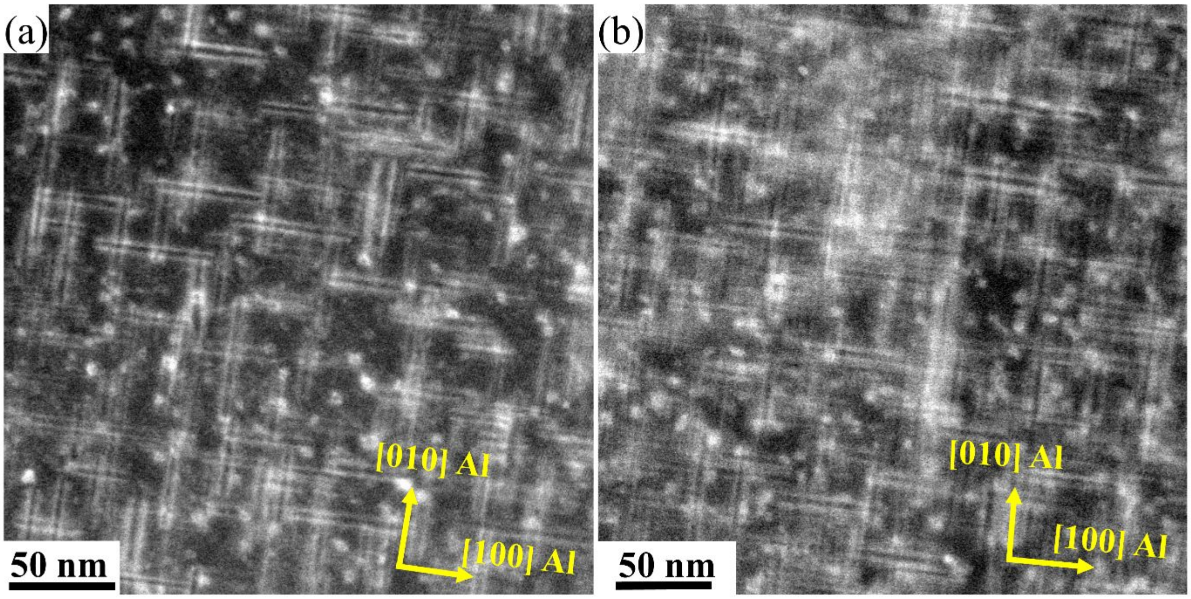

Figure S1. HAADF-STEM images (a, b) of hardening precipitates observed in alloys O2 and O3, respectively.

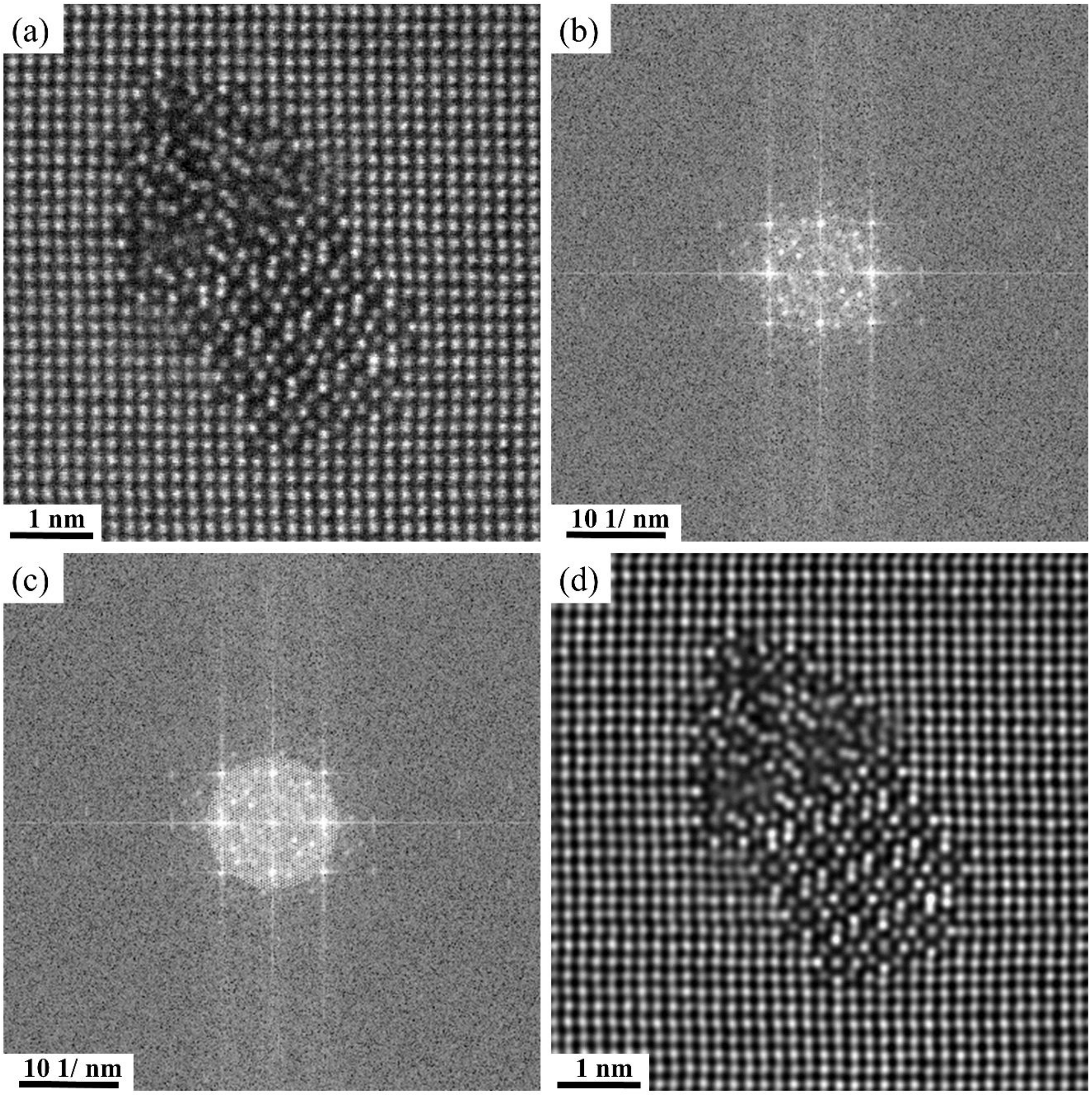

Figure S2. (a) HAADF image of precipitate can be found in alloy O2 in the unfiltered state, (b) Fast Fourier Transformed pattern, (c) application of a band pass mask to filter and remove the noise and (d) filtered HAADF image used for determining the precipitate structures.

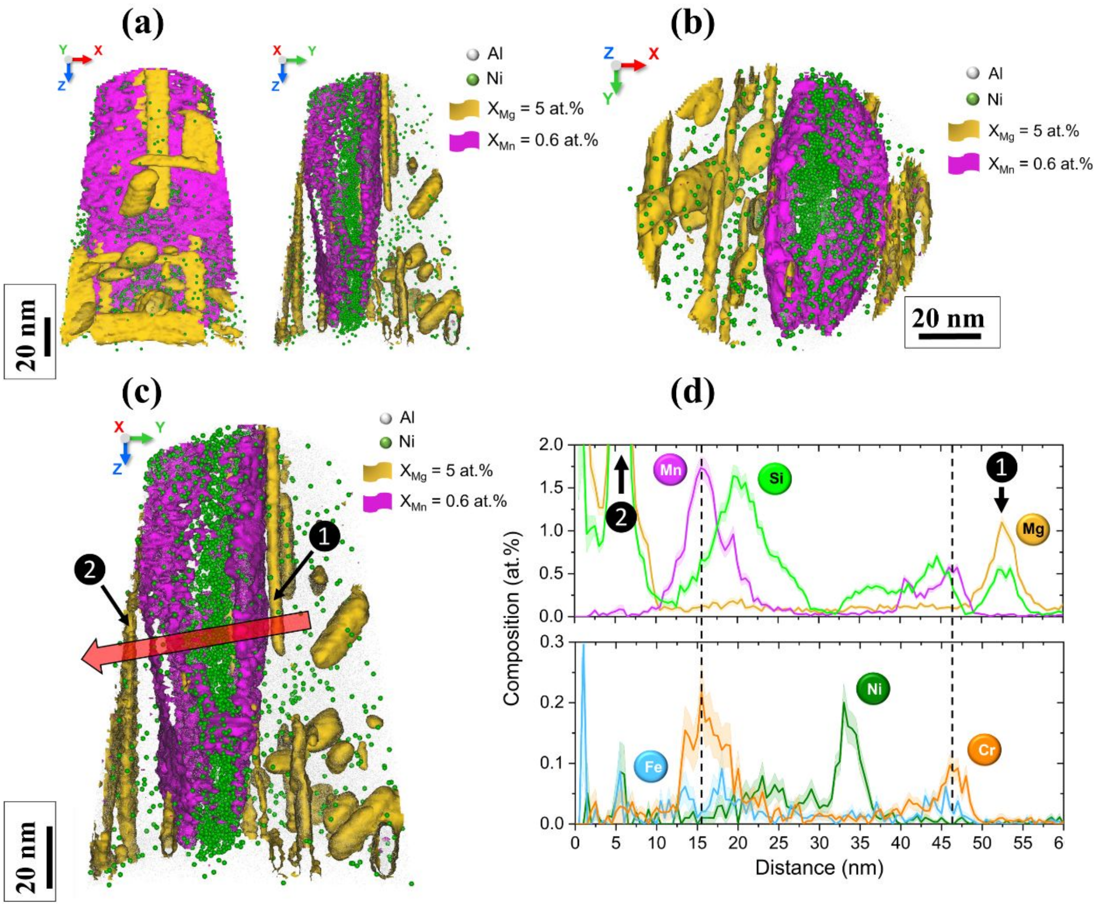

Figure S3. APT analysis of alloy O2: (a), (b), and (c) Atomic reconstruction displaying the precipitates by Mg iso-concentration surface at 5 at.% and dispersoid by Mn iso-concentration surface at 0.6 at.%. (d) Concentration profile of dispersoid type precipitate along the red arrow in (c), see Chapter 11.
